# Development and evaluation of pH-sensitive biodegradable ternary blended hydrogel films (chitosan/guar gum/PVP) for drug delivery application

**DOI:** 10.1038/s41598-021-00452-x

**Published:** 2021-10-28

**Authors:** Zunaira Huma Ghauri, Atif Islam, Muhammad Abdul Qadir, Nafisa Gull, Bilal Haider, Rafi Ullah Khan, Tabinda Riaz

**Affiliations:** 1 Institute of Polymer and Textile Engineering, University of the Punjab, Lahore, 54590 Pakistan; 2 School of Chemistry, University of the Punjab, Lahore, 54590 Pakistan; 3 Institute of Chemical Engineering and Technology, University of the Punjab, Lahore, 54590 Pakistan

**Keywords:** Chemistry, Materials science

## Abstract

pH responsive hydrogels have gained much attraction in biomedical fields. We have formulated ternary hydrogel films as a new carrier of drug. Polyelectrolyte complex of chitosan/guar gum/polyvinyl pyrrolidone cross-linked via sodium tripolyphosphate was developed by solution casting method. Fourier transform infrared spectroscopy, scanning electron microscopy and thermogravimetric analysis were conducted to examine the interactions between the polymeric chains, surface morphology and thermal stability, respectively. The swelling tests resulted that the swelling was reduced with the increase in the concentration of crosslinker due to the more entangled arrangement and less availability of pores in hydrogels. Ciprofloxacin hydrochloride was used as a model drug and its release in simulated gastric fluid, simulated intestinal fluid and phosphate buffer saline solution was studied. pH responsive behaviour of the hydrogels have subjected these hydrogels for drug release applications.

## Introduction

In occasion of chronic diseases which are treated by antiseptics, antibiotics and anti-inflammatory drugs, there is a need of effective drug transport mechanism^1^. The use of pH sensitive hydrogels for the slow drug release at the targeted area for the control of infection has gained much attention in biomedical areas^2–4^. Hydrogels made by the physical or chemical interactions of the various functional groups exist in the polymer network are responsible for the hydrophilicity of hydrogels^5,6^. The importance of pH sensitive hydrogels is because of their swelling response in pH of different body organs and fluids^7,8^. The properties of hydrogels like biocompatibility^9^, biodegradability^10^, softness, superabsorbancy^11^ and mechanical strength^12^ make them useful in different biomedical fields^13^. Hydrophilic groups, cross-linking density and swelling media control the swelling ratio of the hydrogels^14^. Hydrophilic groups are also responsible for the interactions of hydrogel with the biological tissues^15,16^. Hydrogels are advantageous for biomedical applications due to their easy encapsulation of the drug, equal distribution within an irregular shaped defects and also permitting to remain at specific site^17,18^.

Hydrogels can be fabricated by cross-linking the natural polymers but they have weak mechanical strength^19^. Synthetic polymers can be added in a small amount to achieve significant mechanical strength of the hydrogels to reach the targeted area for the drug release^20,21^. Chitosan is a natural polysaccharide obtained by the deacetylation of chitin^22^. Main sources of chitin are crustacean shell creatures like prawns, crabs, shrimps, ants, etc.^23^. It is a suitable material for the applications of drug delivery because it is degraded by the enzymes, less toxic and has low mechanical integrity^24–26^. The presence of amine groups in the chitosan structure enables it to respond against different pH^27^. Guar gum, a natural polysaccharide has an extensive use in developing hydrogels due to its invasive swelling and hydrophilic nature which help it in the rapid drug release^28^. It is used with pH responsive polymers and explored for their competency in targeted drug delivery^29,30^. Pure polysaccharide hydrogels have very low mechanical strength and break quickly in the fluid system resulted in a burst drug delivery^31^. To enhance the mechanical strength, synthetic polymers like polyethylene glycol, polyvinyl pyrrolidone (PVP) and polyvinyl alcohol etc. can be added to the hydrogel blends^31^. Blending is the mixing of different polymers to achieve desirable properties^32,33^. PVP, a synthetic organic polymer, is hydrophilic, biocompatible, low cytotoxicity and its blending with natural polymers gives desirable characteristics to hydrogels for controlled drug delivery^34^.

Ciprofloxacin hydrochloride (CFH) is an antibiotic drug which is absorbed rapidly in stomach after its oral intake. CFH releases in different solutions having different pH values indicated that there must be some pancreatic secretions for its maximum release^35^. Capsules, tablets and injections of CFH have been enormously used but usual methods of administration have its bioavailability of only 52%. So that, half-life for CFH is very short to attain sustained drug delivery^36^.

Butt et al. developed pH-sensitive hydrogels by using natural and synthetic polymers; chitosan, guar gum and polyethylene glycol which were crosslinked by silane groups of tetraethyl orthosilicate and achieved its hydrophilicity and specificity for drug delivery^37^. Gierszewska et al. synthesized pH responsive hydrogels based on chitosan and alginate which were cross-linked through sodium tripolyphosphate (Na-TPP) for the targeted drug release profile^38^. Hanna et al. prepared hydrogel for the sustained release of ciprofloxacin HCl using N-trimethyl chitosan and sodium carboxymethyl xanthum gum^39^.

The main interest of the present study was to develop novel Na-TPP cross-linked pH sensitive ternary blended hydrogel films using biocompatible and biodegradable natural and synthetic polymers; chitosan, guar gum and PVP. The combined properties of these three different polymers have the ability for controlled drug delivery. To the best of our knowledge, this formulation has not been reported yet for the delivery of CFH. The main reason for using Na-TPP was its non-toxicity to the living cells. The effect of using different amounts of crosslinker on the properties of the developed hydrogels was evaluated. Swelling tests were performed in water, buffers and electrolyte solutions. CFH was loaded to the hydrogel and its release mechanism was examined in simulated gastric fluid, simulated intestinal fluid and phosphate buffer saline solution using UV–visible spectroscopy.

## Results and discussion

### Fabrication of hydrogels

Chitosan (0.7 g) was dissolved in 40 mL of 1% aqueous solution of formic acid at 60 °C along with constant stirring on hot plate (Wisd laboratory instruments; Model: MSH 20A) for 1 h. Guar gum (0.2 g) was dissolved in 20 mL of distilled water and stirred for 1 h at room temperature. Both (guar gum and chitosan) solutions were added together and stirred for 1 h. PVP (0.1 g) solution was prepared in 5 mL of distilled water and added to the mixture and continued the blending for 1 h at 60 °C. Na-TPP (crosslinker) was added in the mixture and continued stirring for 3 h at 60 °C. The prepared blend was poured in dry petri dishes and dried in oven at 65 °C. After that, hydrogel films were carefully peeled off from petri dishes after complete drying. These dried films were washed with ethanol to remove unreacted crosslinker and acid contents from the surface of the films. Finally, the prepared hydrogel films were stored in polythene bags in a desiccator. CGP was the controlled hydrogel contained chitosan, guar gum and PVP only, while CGP0.1, CGP0.07 and CGP0.05 hydrogels comprised of 0.1, 0.07 and 0.05% of Na-TPP with the same amount of chitosan, guar gum and PVP, respectively. This ratio of polymers was used to check the effect of crosslinker on properties of hydrogels. The codes and concentrations of the developed hydrogels are provided in Table 1.

**Table 1:** Sample codes along with the quantities of components of hydrogels.

Sample code	Chitosan (g)	Guar gum (g)	PVP (g)	Crosslinker Na-TPP (%)
CGP	0.7	0.2	0.1	0
CGP 0.1	0.7	0.2	0.1	0.1
CGP 0.07	0.7	0.2	0.1	0.07
CGP 0.05	0.7	0.2	0.1	0.05

**Figure 1:**
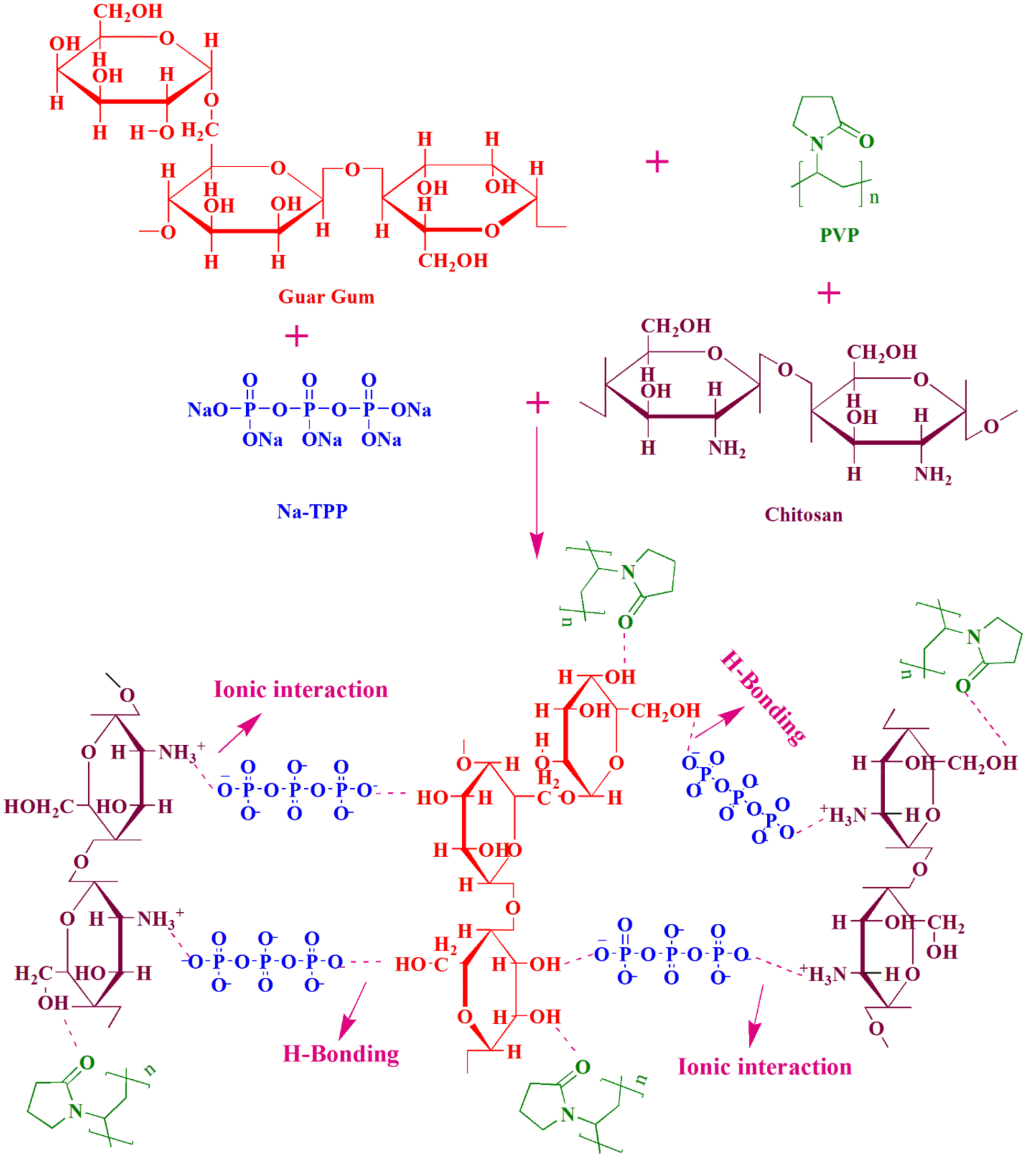
Interactions between the components of CGP hydrogel.

The proposed interactions (physical and chemical) between the components of ternary blended hydrogel films are given in Fig. 1 and the overall pictorial view of the research work is given in Figure S1. As shown in the Figure, chitosan, guar gum, PVP and Na-TPP have physical and chemical electrostatic interactions provided the hydrogel with a stable structure. The protonation of amino groups of chitosan in acidic media made them positive ions and they developed an ionic attraction with the negative charged phosphoric ions of Na-TPP^38,40,41^. The –OH groups of guar gum formed hydrogen bonding with the cross-linker and PVP and hence, formed the efficient complex crosslinked structure. These electrostatic interactions among polymer chains made them stimuli responsive because of which they could be considered as suitable candidates for the controlled drug delivery.

### FTIR spectroscopy

**Figure 2:**
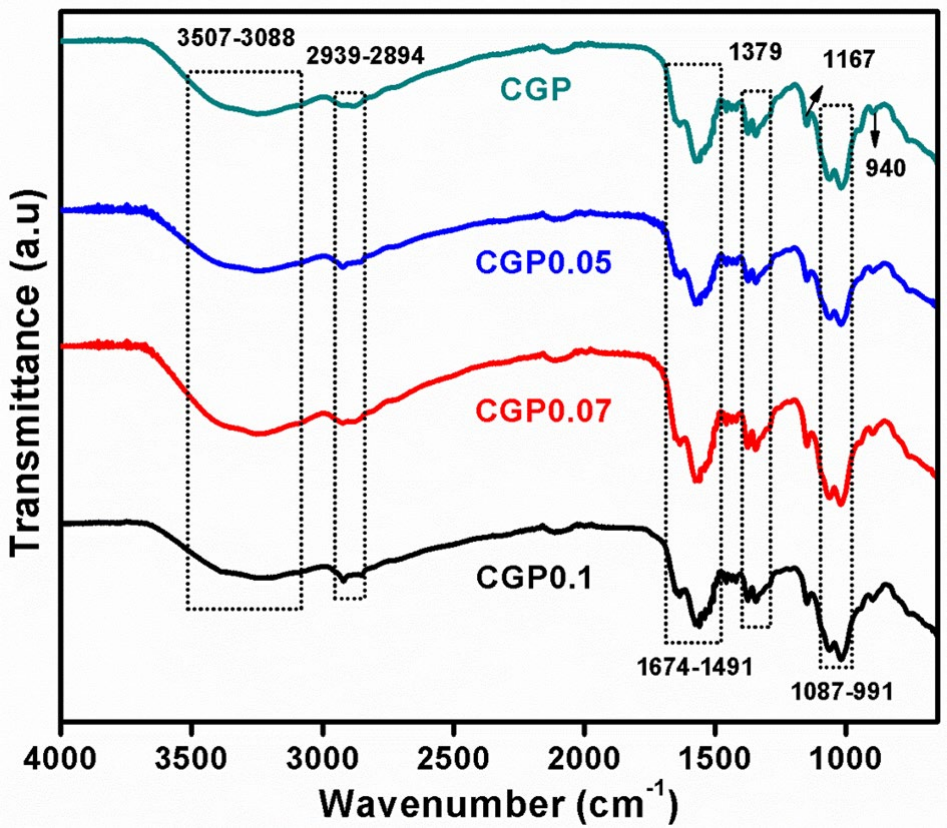
FTIR spectra of CGP hydrogels in the range of 4000–650 cm^−1^.

FTIR spectra of all the ternary blended hydrogel samples are given in Fig. 2. The absorption peaks for chitosan were observed at 1379, 1491 and 1674 cm^−1^ confirmed the presence of cis-amide III, amide II and amide I groups, respectively. A peak observed at 1648 cm^−1^ confirmed the bending vibrations of –OH groups of guar gum and chitosan. A peak at 1167 cm^−1^ was due to the existence of pyranose and saccharine rings of guar gum and chitosan, respectively. Peaks at 1293 and 1019 cm^−1^ showed the presence of cyclic –C–O–C– and acyclic –C–O–C– of guar gum and chitosan, respectively. Peaks at 2939–2894 cm^−1^ were observed which showed the existence of –CH stretching. A broad band was observed at 3506–3088 cm^−1^ indicated the inter- and intra-molecular hydrogen bonding between the polymer chains. An intense peak was observed at 1087–991 cm^−1^ indicated the existence of P=O and P–O vibrations^40,42^. A region at 1200–1000 cm^-1^ implies for the C–N stretching and a peak at 1379 cm^−1^ showed the existence of C–H vibrations of PVP^43^. A region at 1600–1500 cm^−1^ was possibly due to the ionic interaction of negatively charged phosphate groups of Na-TPP and positively charged amino groups of chitosan^42^.

### Swelling studies

#### Swelling in distilled water

**Figure 3:**
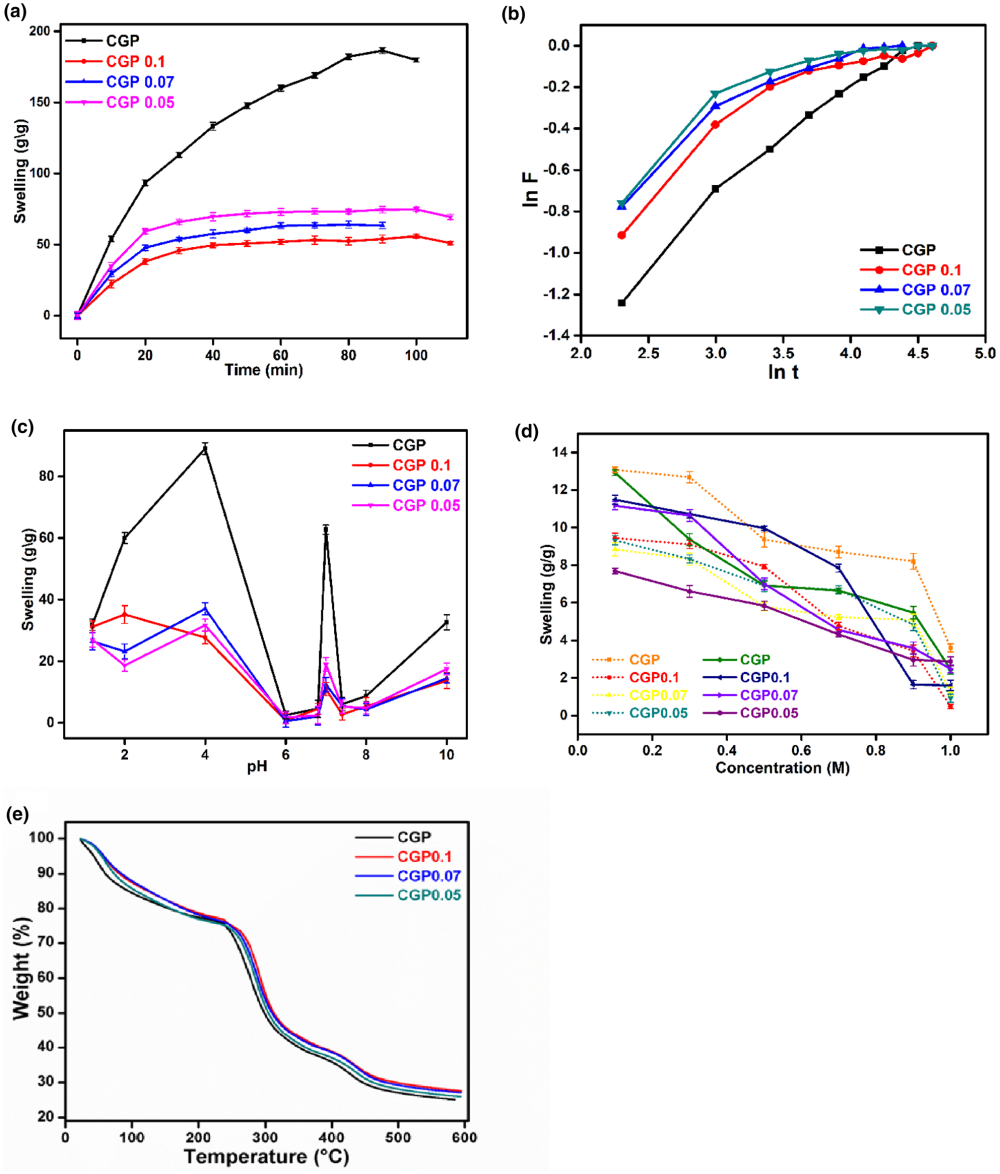
(**a**) Time dependent swelling behavior of CGP hydrogels in distilled water. (**b**) Kinetics of CGP hydrogel samples in distilled water (**c**) Swelling response of CGP hydrogels in different pH solutions (**d**) Swelling response of CGP hydrogels in different molar concentrations of NaCl (dotted) and CaCl_2_ (solid) (**e**) TGA thermograms of CGP hydrogels. Data is presented as a mean ± SD (n = 3).

Figure 3a shows the swelling of the hydrogels in water with respect to time. Swelling of all the hydrogel samples linearly rose with respect to time but all the samples gained their equilibrium at dissimilar points. It is clearly observed from Figure that crosslinker had a negative effect on swelling behavior. Swelling decreased with the addition of crosslinker to the sample. CGP0.1 has shown maximum swelling time of 100 min and its swelling was 55.8 g/g while minimum swelling time was displayed by CGP0.07 of 80 min and its swelling value was 64.04 g/g. CGP displayed the highest swelling (186 g/g) at an equilibrium time of 90 min because it had no crosslinker and its structure was less denser than other crosslinked structures. CGP0.05 exhibited an equilibrium time of 100 min, and among the crosslinked samples it showed maximum swelling tendency. Addition of crosslinker led to increase in the pore numbers in the polymeric structure but the pore size reduced and water entry in the structure was prohibited.

#### Swelling kinetics

Swelling of the hydrogel films depends on the distribution of the solvent from extracellular matrix to its structure. The swelling data was validated by using Ritger–Peppa’s equation as given in Eq. (1).

1 $${\text{F}} = k{\text{t}}^{n}$$

where *k* represents the rate constant for swelling, F is the fractional swelling calculated by dividing Weq and Wt, Weq is the swelling at equilibrium time and Wt is the swelling at time t. Swelling data of hydrogels in distilled water was used to calculate the values of ‘*k*’ and ‘n’ and the value of ‘n’ defines the transport pathway of solvent in hydrogels. If n ≤ 0.5, transport mechanism is indicated as Fickian but if n ≥ 0.5 but less than 1, transport mechanism is non-Fickian. For the developed hydrogels, a plot between ‘ln t’ and ‘ln F’ is shown in Fig. 3b by using their swelling values in water. The calculated values for diffusion parameters are also given in Table 2. From data, it was concluded that developed hydrogels showed Non-Fickian diffusion mechanism. The relaxation rate was higher while diffusion rate was lower in transport of solvent.

**Table 2:** Diffusion parameters of CGP hydrogel samples.

Parameters	CGP	CGP0.1	CGP0.07	CGP0.05
*N*	0.55	0.51	0.58	0.59
Intercept	− 2.414	− 2.027	− 2.21	− 2.1
*k*	0.089	0.132	0.109	0.122
Regression (%)	97.8	91.72	94.71	90.66

#### Swelling in different buffers

Different buffers having pH (10, 8, 7.4, 7, 6.8, 6, 4, 2, and 1.2) were prepared to study the response of developed hydrogel films. The swelling results of developed hydrogel films in different buffers against time are shown in Fig. 3c. All the samples displayed maximum swelling at acidic pH and lower swelling at neutral pH while at pH = 10, hydrogels again started to swell. Amongst all the samples, maximum swelling was shown by CGP. This enormous increase in swelling of uncrosslinked hydrogel sample is due to the widest pockets of polymer network. In the crosslinked hydrogel samples, this network become dense and compact due to more covalent and physical crosslinks which ultimately leads to the less swelling. At pH = 7.4, all the samples showed a slight increase in their swelling. The interacting species in the developed hydrogels are –NH_2_ groups. The swelling in acidic media was due to the protonation of amino groups (–NH_2_) of the chitosan to the ammonium ions (–NH_3_^+^). There was also a charge repulsion and decrease in osmotic pressure at lower pH due to the presence of more ionic groups (ammonium ions), caused increase in swelling^38^. When pH was increased, there was a deprotonation of ammonium ions which led to the decrease in swelling due to less polymer chains interactions.

#### Swelling in electrolytes

Swelling test was also performed in different concentrations of electrolytes i.e. NaCl and CaCl_2_. Both salts contain same anion but their cations are different. Na^+^ is monovalent while Ca^+2^ is divalent and these electrolytes are present in a human body. The swelling results of the developed hydrogels in different molar concentrations are shown in Fig. 3d. The results indicated that swelling decreased with increase in the molar concentration of electrolytes. The decrease in swelling was due to the screening effect produced by the surplus charges in the electrolyte solution^44^. An increase in molar concentration of the electrolytes also lower the osmotic pressure difference between hydrogel matrix and electrolytes which hindered the permeation of water into the hydrogel matrix^45^.

### SEM analysis

**Figure 4:**
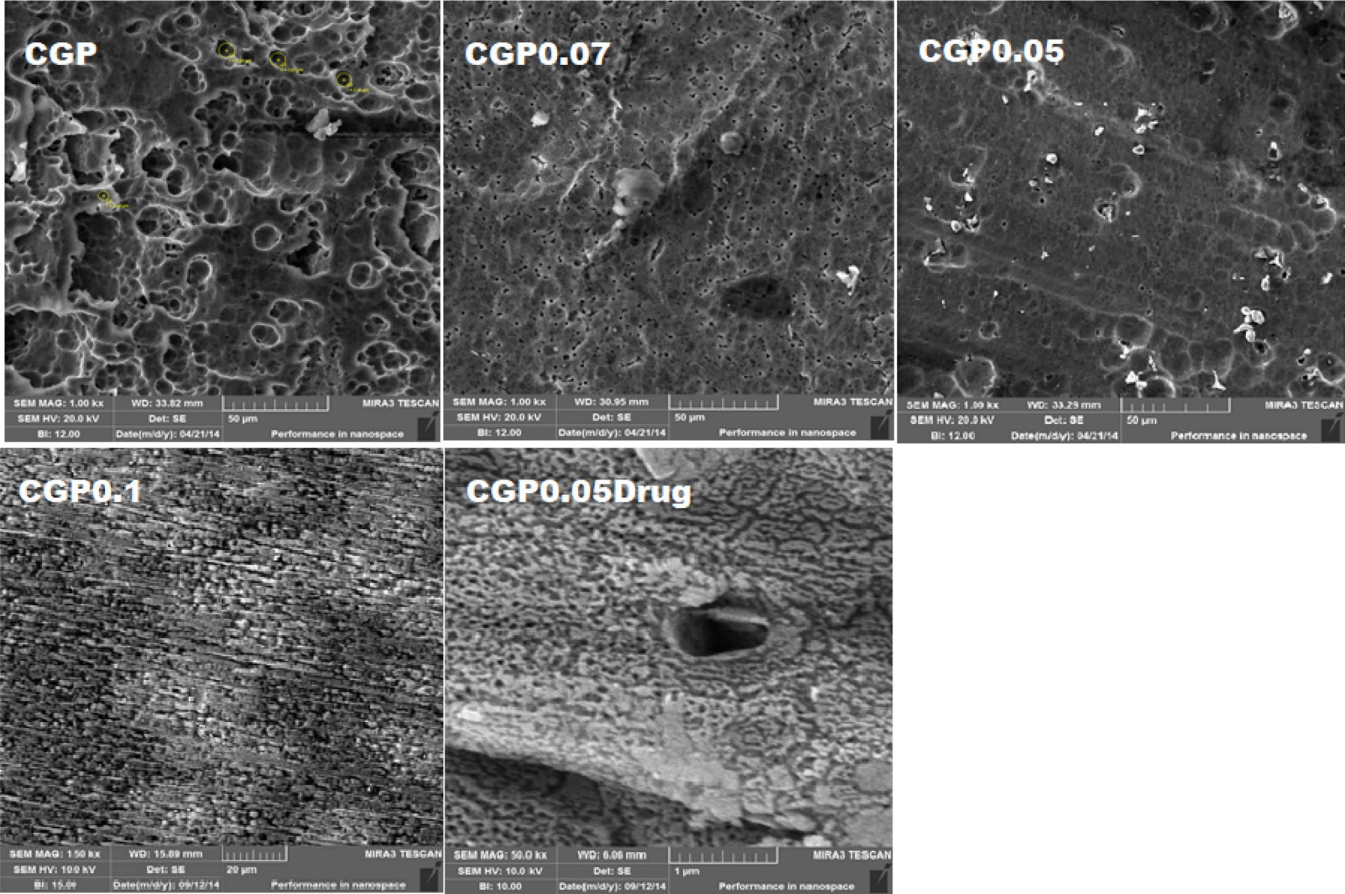
SEM micrographs of CGP hydrogel films.

SEM was conducted to examine the surface framework of the ternary blended hydrogels to analyze the porous network of the hydrogel films and the effect of crosslinking on the polymeric network structure. Figure 4 shows the micrographs of the hydrogels with different concentrations of crosslinker and drug loaded hydrogel. The micrographs indicated the presence of hollow cavities in the hydrogel structure. The comparison of these micrographs revealed that crosslinking decreased the pores in hydrogel structures. The micrograph of drug loaded hydrogel revealed the drug entrapment in the network of hydrogel structure.

### Thermogravimetric analysis

Figure 3e represents the thermograms of CGP hydrogel samples. TGA was conducted to evaluate the influence of crosslinker on the thermal stability of hydrogels as a function of temperature. Figure showed three stages of degradation; first stage inferred to the weight loss from 24 to 240 °C due to vaporization of moisture and bound water. In the second stage, onset of degradation in which the main backbone of the hydrogel degraded started around 240 °C. The offset of degradation was observed around 350 °C. Initially, weak interactions among components of the hydrogels started to break in the second stage of degradation and in the final stage corresponding to the 350–600 °C, polymer chains broken into smaller fragments^46^. It was clearly observed from Figure that crosslinking has a positive effect on the hydrogel framework. More concentration of cross-linker led to more thermally stable hydrogel network system.

### Antibacterial study

**Figure 5:**
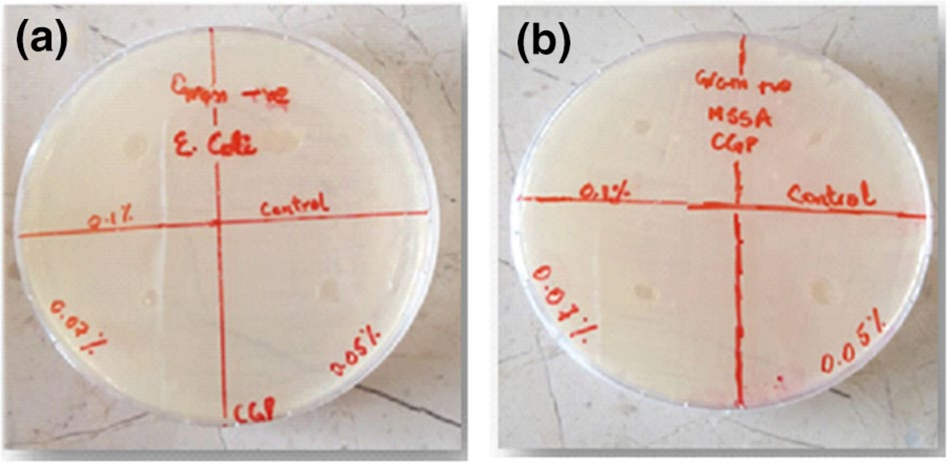
Digital photographs of antibacterial activity of CGP hydrogels (**a**) against *E. coli* (**b**) against *S. aureus.*

Antibacterial performance of the developed hydrogels against different strains of bacteria is given in Fig. 5. Disc diffusion method was used to investigate the antibacterial activity of the developed hydrogel samples against gram negative (*E. coli*) and gram positive (*S. aureus*) strains of bacteria. All hydrogel films showed bacteriostatic behaviour against both strains of bacteria. The developed hydrogels neither allowed the further growth of bacteria nor harmed them but they kept them in a stationary phase of growth. *E. coli* comprises of lipopolysaccharides and phospholipids which gives it a negatively charged cell wall whereas *S. aureus* comprises of an outer covering of peptidoglycolipids and mucopeptide giving it a strong positively charged cell wall^47^. The hydrogel complex has both positive ends (ammonium ions) of chitosan and negative ends (phosphoric ions) of Na-TPP in its structure. Due to the presence of these interactive sites in bacteria and hydrogel system, there might be some interactions among these charged entities which stopped the further growth of bacteria. The phosphate ions of Na-TPP may affect the production of adenosine triphosphate which may cause disturbance in the interconversion cycle of bacteria and the further growth is prohibited^48^. The activity against CGP0.05 drug loaded sample was also performed against both strains.

### Drug release analysis

**Figure 6:**
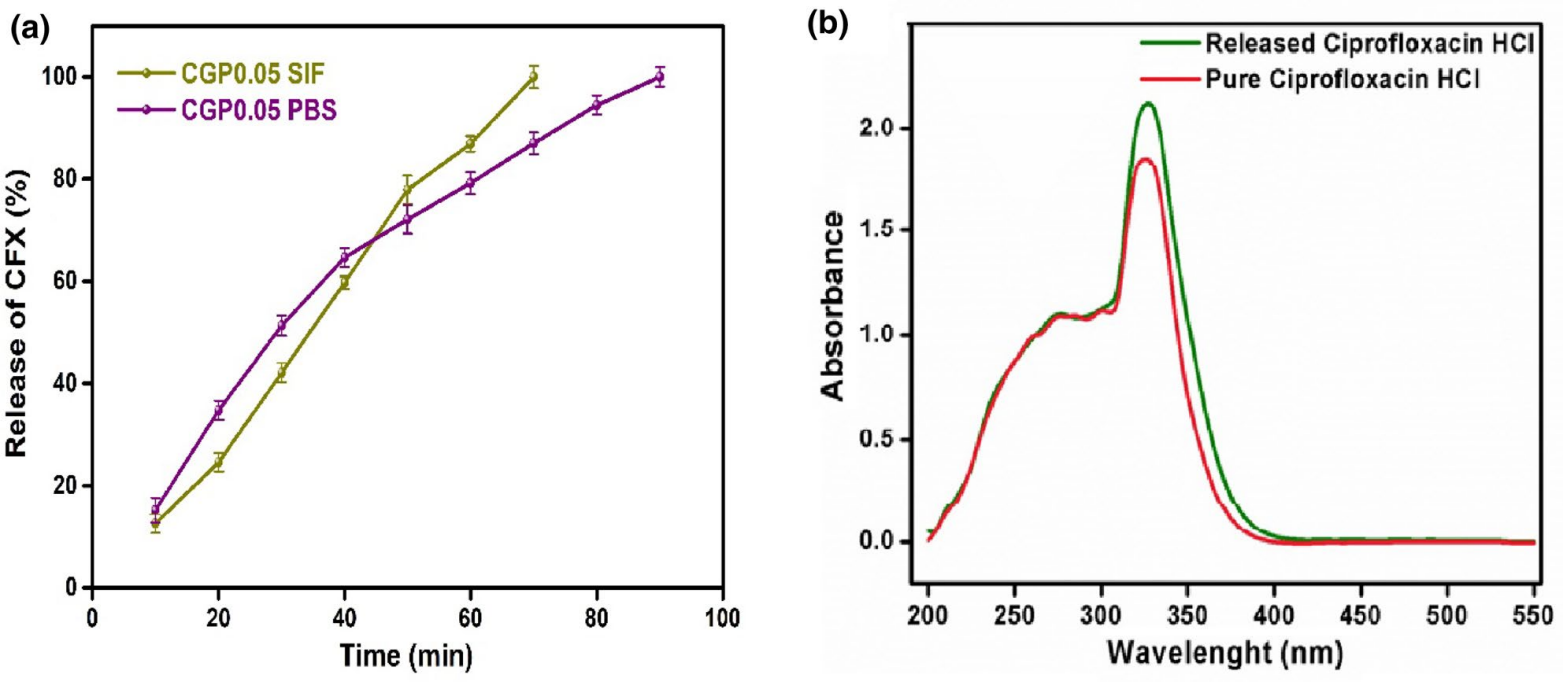
(**a**) Release analysis of CFH in SIF and PBS solutions with time. Data is presented as a mean ± SD (n = 3) (**b**) Chemical activity of pure ciprofloxacin HCl and after release from hydrogel.

CGP 0.05 was nominated for the drug loading because it showed the highest swelling in water. CFH (model drug) was loaded on CGP0.05 and its release profile was checked in phosphate buffer saline (PBS), simulated intestinal fluid (SIF) and simulated gastric fluid (SGF) solutions. The release profile of CFH is shown in Fig. 6a. CFH is quinolone used in this study has hydroxyl, carboxylic acid cyclopropyl and piperazin-1-yl groups that develop physical and chemical interactions among components of hydrogel. It is second generation fluoroquinolone antibiotic that bind and inhibit bacterial DNA. The drug released in the first 30 min was 30% in SGF as it was cleared from swelling studies that hydrogels showed higher swelling at acidic pH. Drug release must be lesser than 10% in SGF in first 30 min according to US Pharmacopeia, indicated that the developed hydrogels cannot be subjected to oral administration of the drug^49^. In SIF and PBS, a sustained release of the drug was observed as shown in Figure. Almost 100% drug was delivered out from the hydrogel in 70 min in SIF while in PBS solution, total drug was released in 90 min. The results presented that the developed ternary blended hydrogel films showed sustained drug delivery and can be administered for intravenous drug release administration.

### Chemical activity of drug

The maintenance of chemical structure of drug after its release from the hydrogel is an important factor. Most of the time, drugs denature after their release from the hydrogel by reacting with hydrogel components and cause some major issues^50^. The drug transporter should be able to transport drug without causing any change in its structure^51^. UV–Vis spectrophotometer was used to observe the chemical activities of pure CFH and the released drug from hydrogel at λ_max_ of 272 nm (Fig. 6b). The comparison of the spectra of both drugs showed that there was no major change in the chemical structure of released drug. The released drug kept its chemical structure after its release.

## Conclusion

pH sensitive novel ternary blended hydrogel films were prepared successfully using chitosan, PVP and guar gum which were cross-linked via Na-TPP with different concentrations. FTIR analysis confirmed the existence of integrated functional groups and connections among components of the blend. The swelling tests in water showed the decrease in swelling with increase in the amount of crosslinker whereas swelling in different pH inferred that hydrogels swollen more at acidic pH while less swelling was experimented at neutral and basic pH. The surface morphology results confirmed the presence of porous structures and the drug (CFH) entrapped in the pores of hydrogels. TGA confirmed the thermal stability of hydrogel samples at different temperatures. The bacteriostatic activity was showed by all hydrogel samples against *E. coli* and *S. aureus.* The drug release profile of CFH showed that the developed formulation can be considered for release of drug.

## Experimental methods

### Materials

Chitosan (viscosity = 600 cps, Mol. wt. = 221,260.6 g/mol, degree of deacetylation = 85%) was received from MP Biomedicals, guar gum (viscosity = 5000 cps) extra pure food grade was received from Dabur India Ltd, PVP (Mol. wt. = 40,000 g/mol) and formic acid (**≥ **90%) were received from BDH laboratory, England. Sodium tripolyphosphate was purchased from Daken Chemicals, Zhengzhou, China. CaCl_2_ and NaCl were received from Sigma Aldrich St. Louis, Missouri, US. Ciprofloxacin hydrochloride was obtained locally and used as a model drug. All the chemicals were of analytical grade and used as received without any treatment.

### Analyses and characterization

#### Fourier transform infrared (FTIR) spectroscopy

FTIR spectra of all the hydrogel samples were obtained by FTIR spectrophotometer, Model: Tensor li, Bruker, spectra in transmittance mode in the range of 4000–650 cm^−1^ on a resolution of 2 cm^−1^ with 32 scans per spectrum.

#### Swelling studies

Swelling tests of the hydrogels were completed in solvents like distilled water, buffer solutions and electrolytes. Hydrogel samples were cut into small fragments (∼ 25 mg), weighed and submerged in solvents, separately at 37 °C. Excess solvent was wiped off with the help of tissue paper after a specific time and the weight of the swollen hydrogel was determined. Same method was repeated till equilibrium time was achieved when no additional water was absorbed by the hydrogels and the weight started to decrease. Triplicate measurements were taken and their average value was calculated and data was organized. Swelling results were calculated by using the following Eq. (2);

2 $${\text{Swelling}}\left( {{\text{g}}/{\text{g}}} \right) = {{\left( {{\text{W}}_{{\text{s}}} - {\text{W}}_{{\text{d}}} } \right)} \mathord{\left/ {\vphantom {{\left( {{\text{W}}_{{\text{s}}} - {\text{W}}_{{\text{d}}} } \right)} {{\text{W}}_{{\text{d}}} }}} \right. \kern-\nulldelimiterspace} {{\text{W}}_{{\text{d}}} }}$$

where W_s_ represents the weight of swollen hydrogel and W_d_ represents the weight of dry hydrogel. Swelling tests were also performed in different buffer solution (pH 1.2, 4, 6, 6.8, 7, 7.4, 8, 10) while the swelling experiments were also calculated in electrolyte solutions of CaCl_2_ and NaCl at different molar concentrations (1, 0.9, 0.7, 0.5, 0.3, 0.1 M).

#### Scanning electron microscopic analysis

Surface morphology of the hydrogel samples was examined through SEM, Model: JEOL/EO JSM 6480 (LA) Akishima, Tokyo, Japan. The micrographs were obtained at different magnifications. Hydrogel samples were swollen in water then freeze dried in liquid nitrogen to get the porous crosslinked structure through SEM. Thickness of each sample was around 0.2 mm.

#### Thermogravimetry/differential scanning calorimetric analysis

TGA of the hydrogel samples was carried out on SDT build 95 module DSC-TGA standard, USA, with nitrogen flow of 100 mL/min from ambient temperature to 600 °C and rate of heating was kept at 10 °C/min.

#### Antibacterial properties

Disc diffusion method was used to study antibacterial properties against two strains of bacteria i.e. *E. coli* (gram negative) and *S. aureus* (gram positive). Discs of hydrogel samples with 6 mm diameter were taken and placed on the agar plates which were inoculated earlier with bacterial strains at a distance of about 3 cm. The plates were incubated for 24 h at 37 °C.

### Drug release profile

#### Preparation of SIF, PBS and SGF solutions

Phosphate buffered saline (PBS) solution was prepared using KCl (0.2 g), NaCl (8 g), KH_2_PO_4_ (0.24 g), Na_2_HPO_4_ (1.44 g) and some drops of HCl were added to maintain the pH at 7.4. All chemicals were dissolved in distilled water and added together in 1000 mL flask and diluted up to the mark with distilled water. Simulated gastric fluid (SGF) of pH 1.2 was prepared by adding HCl (3.5 mL) and NaCl (1 g) in 500 mL flask and diluting the flask up to the mark with distilled water. Simulated intestinal fluid (SIF) of pH 6.8 was prepared by mixing 118 mL solution of NaOH (0.2 M) and 250 mL solution of KH_2_PO_4_ (0.2 M).

#### Preparation of drug loaded sample

To prepare the drug loaded sample, chitosan (0.7 g), guar gum (0.2 g) and PVP (0.1 g) were blended together to attain a homogeneous blend. 50 mg CFH was dissolved in 5 mL distilled water and added dropwise to the mixture and stirred for 1 h. The Na-TPP (0.05%) was added and stirred for 3 h and poured in petri dish and oven dried at 60 °C.

#### Drug release study

Hydrogel loaded with drug was placed in a beaker having 100 mL PBS solution at 37 ºC. After every 10 min interval, 5 mL solution was drawn with the help of syringe and stored in a vial and the beaker was replenished with the fresh PBS solution to balance the solution volume. The collection of samples was continued for 3 h. The amount of the released CFH by the hydrogels was analyzed spectrophotometrically at 272 nm^52^ by UV–Vis spectrophotometer, Labomed, Inc. UVD-3500 USA. For the release analysis, standard reference (100 ppm PBS) was used.

The same procedure was followed for the drug (CFH) release study in SIF and SGF solutions.

#### Chemical activity of drug

The chemical activity of the pure and released drug was examined using UV–Vis spectrophotometer (UV-2600, Shimadzu, Japan).

## Supplementary Information

Supplementary Information.
