# Supplementary material for: Development and evaluation of pH-sensitive biodegradable ternary blended hydrogel films (chitosan/guar gum/PVP) for drug delivery application
Source: Sci Rep. 2021 Oct 28;11:21255. doi: 10.1038/s41598-021-00452-x (PMC8553746; doi:10.1038/s41598-021-00452-x)
Supplement: Supplementary file 1 — Supplementary Information. [file 41598_2021_452_MOESM1_ESM.doc]

Supplementary Information

**Development and evaluation of pH-sensitive biodegradable ternary blended hydrogel films (chitosan/guar gum/PVP) for drug delivery applications**

Zunaira Huma Ghauri^a^, Atif Islam^a*^, Muhammad Abdul Qadir^b^, Nafisa Gull^a^, Bilal Haider^c^, Rafi Ullah Khan^a^, Tabinda Riaz^a^

^a^Institute of Polymer and Textile Engineering, University of the Punjab, Lahore, 54590, Pakistan

^b^School of Chemistry, University of the Punjab, Lahore, 54590, Pakistan

^c^Institute of Chemical Engineering and Technology, University of the Punjab, 54590, Lahore, Pakistan


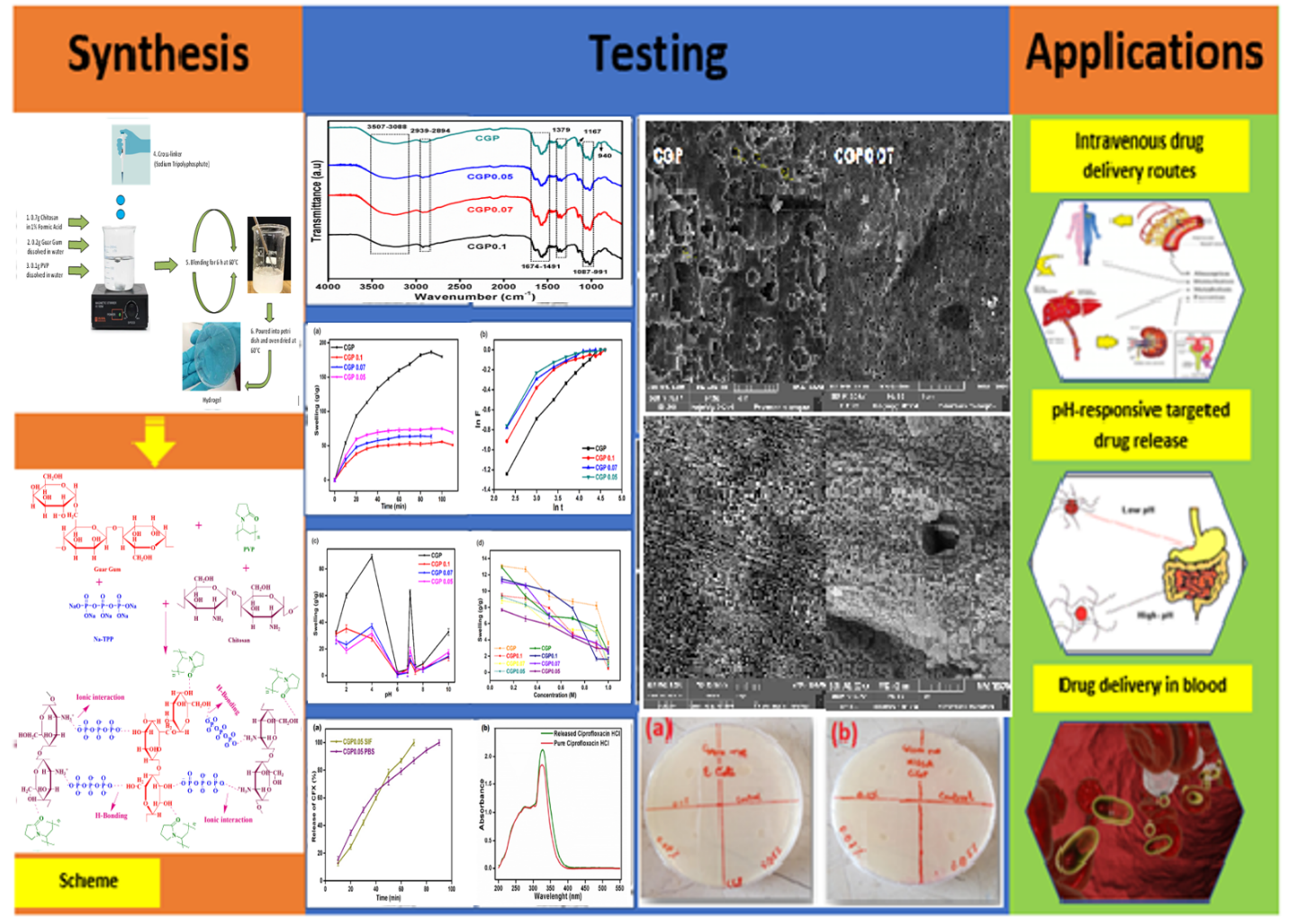


**Figure S1.** Pictorial view of the formulation, testing and applications of CGP hydrogels
